# Incidence of *Diaphorina citri* Carrying *Candidatus* Liberibacter asiaticus in Brazil’s Citrus Belt

**DOI:** 10.3390/insects11100672

**Published:** 2020-10-03

**Authors:** Nelson A. Wulff, Bruno Daniel, Rodrigo S. Sassi, Alécio S. Moreira, Renato B. Bassanezi, Ivaldo Sala, Daniela A. B. Coletti, Júlio C. Rodrigues

**Affiliations:** 1 Fundo de Defesa da Citricultura–FUNDECITRUS–Araraquara, São Paulo 14807-040, Brazil; agrobd@hotmail.com (B.D.); renato.bassanezi@fundecitrus.com.br (R.B.B.); ivaldo.sala@fundecitrus.com.br (I.S.); daniela.coletti@fundecitrus.com.br (D.A.B.C.); julio.rodrigues@fundecitrus.com.br (J.C.R.); 2 Professional Master Program on Citrus Diseases and Pest Control (MasterCitrus)/FUNDECITRUS–Araraquara, São Paulo 14807-040, Brazil; Rodrigo.Sassi@citrosuco.com.br; 3 Embrapa Cassava & Fruits, Cruz das Almas BA 44380-000, Brazil; alecio.moreira@embrapa.br

**Keywords:** Asian citrus psyllid, huanglongbing, edge effect, yellow sticky traps, qPCR, management, *Candidatus* Liberibacter

## Abstract

**Simple Summary:**

*Candidatus* Liberibacter spp. associated with citrus huanglongbing (HLB) comprise species *Ca.* L. africanus (Laf), *Ca*. L. asiaticus (Las), and *Ca*. L. americanus (Lam). While Laf is vectored by the psyllid *Trioza erytreae*, Las and Lam are vectored by *Diaphorina citri*. Las spread by *D. citri* is a major factor for the incidence of HLB-affected citrus in Asia and South, Central, and North America. Recently, both *D. citri* and Las were also detected in continental Africa, while *T. erytrea* was found in Portugal and Spain. *D. citri* that feed as nymphs in citrus harboring Las are better vectors when become adults than adults that acquire Las. Planting nursery trees free of *Ca*. Liberibacter spp., removing diseased symptomatic citrus trees, and controlling the psyllid population are management practices able to keep infection at acceptable rates. Psyllid population levels, as well as the percentage of individuals that carry Liberibacter, are important factors to succeed against HLB. Area-wide HLB management has been used in Brazil since 2009 and, in this context, psyllid population increase is monitored for control. The same integrated system was used to collect psyllids from traps over a three-year period, as a large-scale resource to address the effect of local management on the percentage of *D. citri* harboring Las. Good HLB management practices resulted in lower numbers of psyllids with Las. Location and regional disease incidence also affected the percentage of psyllids with Las, ranging from 33 to 74.6%.

**Abstract:**

Huanglongbing (HLB) is a citrus disease of worldwide importance, associated with the presence of *Candidatus* Liberibacter asiaticus (Las) and vectored by the psyllid *Diaphorina citri* in Asia and the Americas. To properly manage HLB, removal of inoculum sources and control of the psyllid are undertaken. We evaluated the percentage of the psyllid population with Las, sampled from yellow sticky traps over a three-year period and its relationship with insect population, regions, season of the year, and HLB management in citrus areas in the southwestern, central, and northern regions of São Paulo (SP) and southwestern region of Minas Gerais states, Brazil. In each reading, up to 50 psyllids per region were collected and detection of Las in individual psyllids were made by quantitative polymerase chain reaction. The percentage of psyllids with Las—an average of 65.3%—was constant throughout the year in the southwestern region of SP state, while showing an increase from spring to autumn when sampled from central to northern regions. The proportion of psyllids carrying Las from each region and year period were compared by a proportion test and spectral density analysis. The proportion of psyllids carrying Las evaluated in the same region in different seasons presented statistical differences in central (Araraquara) and southwestern (Santa Cruz do Rio Pardo) regions in 2015, with higher values in the first semester (summer and autumn) than in the second semester (winter and spring). Orchards with poor HLB management had higher incidence of psyllids with Las. Spectral density analysis indicated that good management areas had 50% less relevant peaks of psyllids with Las than in areas with poor HLB management practices. The relationship between the percentage of psyllids carrying Las and the number of captured psyllids in the region in a given time denotes the most critical intake time for HLB spread in citrus orchards. The reduction in the population of psyllids carrying Las is a direct benefit from the use of good management practices.

## 1. Introduction

Citrus is one of the most cultivated fruit trees and Huanglongbing (HLB) is a worldwide disease severely threatening its production and profitability [1,2]. HLB is associated with the Liberibacters, *Candidatus* Liberibacter asiaticus (Las), *Ca.* L. africanus (Laf), and *Ca.* L. americanus (Lam). Las has wider occurrence than Laf and Lam and as in the case of Lam, is transmitted by the psyllid *Diaphorina citri* (Hemiptera: Liviidae) [3,4]. In Brazil, Lam was reported to be the prevalent Liberibacter along with minor presence of Las in 2004 [5,6], however there was a shift in the occurrence of Las and currently, it has, by far, been the most prevalent Liberibacter found in citrus orchards for several years [2]. Since other HLB associated bacteria, such as phytoplasmas [7,8], are not known to be associated with psyllids [9] and Laf is not found in Brazil [10], studies on the interaction between Las and *D. citri* are of capital importance.

HLB management relies on three main tactics, called the three-pronged system (TPS) [11], comprised of planting healthy nursery trees, inspection followed by eradication of symptomatic trees, monitoring, and control of insect vectors. By applying TPS in HLB management, several farms were able to cope with HLB, though to some extent, these factors lie outside the farms [12]. To account for inside and outside citrus orchard factors, the strategy of regional HLB management was developed to cover larger areas [13]. In this context, to monitor the regional occurrence of psyllids [14] is a key factor to control the spread of the HLB vector alongside the primary dissemination of *Ca.* Liberibacter spp. [13]. Indeed, management by the TPS system is widely applied in São Paulo State and Triângulo Mineiro/Southwest of Minas Gerais State citrus belt, resulting in disease incidence averages from 17.89 to 20.87% of infected trees between 2015 to 2020 [15].

The dispersal of psyllids with Las is a source of HLB introduction into orchards. The presence of Liberibacter in *D. citri* is correlated with the primary spread of HLB [13]. *D. citri* has frequent movement between groves, with a greater number of adults moving from unmanaged into managed plots than the opposite [16]. The dispersal capacity of *D. citri* is greater in the absence than in the presence of young leaves [17]. Temperature is a major factor to flight initiation but air humidity and host characteristics might influence the behavioral responses of *D. citri* as well [18], such as the presence of young shoots [17], particularly the ones most adequate for oviposition [19].

Nymphal acquisition of Las results in higher risk infective vectors than adult acquisition [20] and indeed, the ability of *D. citri* nymphs to acquire Las at the asymptomatic stage, a few days after inoculation, seems to be key in the dissemination of the bacterium [21]. This implies that psyllid monitoring and knowledge of the presence of Las in the psyllid are important factors to understand the epidemiology of the disease and to better implement management strategies to cope with HLB.

Detection of Las in *D. citri* is carried out mainly with qPCR, targeting the 16SrDNA [22,23] or *tuf*B [20] and the bacterium is able to infect the whole psyllid body [24,25]. Las prevalence in *D. citri* in Mexico was 58.2% in the Colima region [26]. Seasonal variability in the proportion of positive adults was observed, whereby winter and spring had the highest levels. This season marks the co-occurrence of a high density of potentially infective psyllids on a susceptible host with the continual generation of new shoots and optimal environmental conditions [26]. In Florida, a 17.5% incidence was reported [27], generally higher during late fall or early winter and often lower during mid-to-late summer, with a significant negative correlation between psyllids with Las and air temperature. Transmission rates might be higher during periods of higher *D. citri* infestations [27]. Reports of Las incidence in psyllids in Brazil are restricted to field assays, varying from 1.1 to 14.2% [13] and the current survey was carried out in a statewide range to uncover the prevalence of Las in *D. citri* psyllids caught in sticky traps.

## 2. Materials and Methods

### 2.1. Locations, Adult Psyllid Monitoring, and Sample Collection

**Figure 1:**
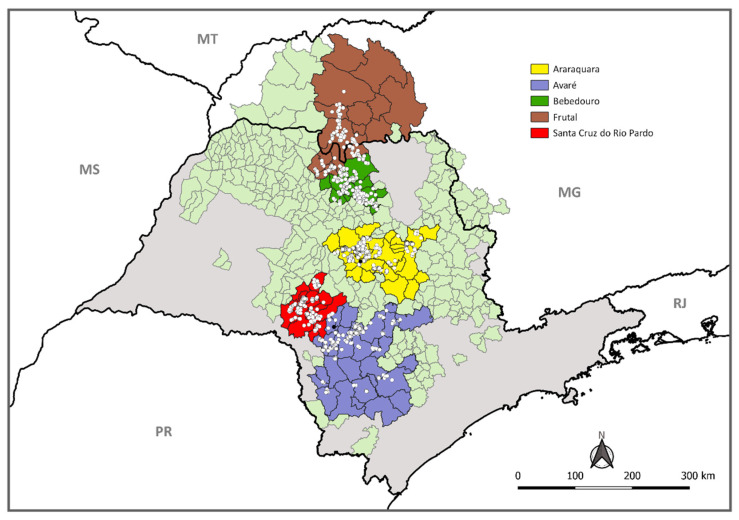
The map illustrates in light green the municipalities having commercial citrus in the states of São Paulo (SP) and Minas Gerais (MG), with municipalities belonging to each region colored: southwestern (Avaré and Santa Cruz do Rio Pardo), central (Araraquara), northern (Bebedouro) of SP state, and northern SP/southwestern of MG state (Frutal). White dots represent places where yellow sticky traps had psyllids sampled for the detection of *Candidatus* Liberibacter asiaticus. Black dots are the two farms where traps from HLB management type A, in Avaré and Araraquara regions, were sampled.

Yellow sticky traps monitored by Fundecitrus as part of the Psyllid Alert System [28] from southwestern, central, and northern regions of São Paulo state (SP) and the southwestern region of Minas Gerais state (MG) are placed in poor HLB managed areas. Traps monitored by the growers from two farms, one from the southwestern (Iaras) and another from central SP (Gavião Peixoto), with strict HLB management practices, comprised the sampling scheme. The average number of traps monitored for *D. citri* sampling per region is indicated in Supplementary Table S1. Traps were placed at the edge of citrus farms or citrus backyard trees, in the superior third part of a citrus plant, facing the exterior of the orchard. Each trap was read and changed fortnightly by trained inspectors for the presence of psyllids. Each reading for a given fortnightly period was a single time point. The location of monitored traps is indicated in Figure 1. The detailed global positioning system from the sampled traps is given in Supplementary Table S2. For each region, up to 50 psyllids were randomly collected from the monitored traps set, with a maximum of two psyllids per trap per site. Each individual psyllid was removed from the yellow sticky trap no longer than three days after the trap was collected in the field, with the help with a steel wire. Each psyllid sample was stored in 70% ethanol in an individual tube. Transport was under an ambient temperature and delivered for processing at the Diagnostic Laboratory at Fundecitrus, Araraquara–SP, where samples were stored at −20 °C until further processing.

### 2.2. HLB Management and Citrus Shoot Stage Evaluation

Citrus farms that apply strict HLB management practices based on TPS [11] were called type A management, which was the only category with HLB symptomatic tree eradication. When symptomatic tree eradication was not performed, but intense psyllid control was done, there was type B management. Management A and B have strict psyllid control in common where insecticides are applied at least once a month. When only occasional psyllid control was carried out, without inoculum reduction by HLB tree eradication, there was type C management. During trap readings at fortnightly intervals, inspectors also evaluated the prevalent flush shoot stage of citrus plants in the tree the trap was placed. The number of trees with prevalent vegetative (V) flush in stages V1 (dormant and swelling buds), V2 (adaxial new leaf surfaces not visible), and V3 shoots (adaxial new leaf surfaces visible and unfolded) [19] were added and divided by the total number of trees evaluated.

### 2.3. Detection of Candidatus Liberibacter asiaticus by qPCR

Each psyllid was removed from 70% ethanol and air dried. Sample crushing was done manually with the aid of a plastic pistil for samples collected from 2014 to mid-2016 and from mid-2016 to 2017, samples were processed in a Tissue Lyzer (Quiagen, Heiden, Germany) using a single stainless-steel bead. Total DNA was extracted from single psyllids and analyzed in a duplex qPCR without prior DNA measurement and essentially as described [29], for the presence of the 16SrDNA from Las with HLBaspr primer/probe (5′FAM/BHQ1, Macrogen, Seoul, Korea) [22] and *D. citri wingless* gene (DCp; 5′HEX/BHQ1, Macrogen) [23]. The quantification cycle threshold (Ct) was manually adjusted using the StepOnePlus software version 2.3 (ThermoScientific, Waltham, MA, USA) for the target sequences. All psyllids collected from the traps had DNA extracted and duplex qPCR performed. Samples without detectable levels of the *D. citri wingless* gene, i.e., Ct values equal or above 36 were removed from further analysis. Percentage and proportion analyses were carried out only with samples with positive values for *D. citri*. The remaining samples were considered positive for the presence of 16SrDNA from Las when Ct values were equal or below 35.0 and negative when above this cut-off value. Titer was measured only in samples falling below this limit. Standard curves for *D. citri wingless* genes were generated with standard procedures after amplicon cloning, while for Las we used published data [30]. Ct limits were assessed with serial dilutions of positive samples and cloned amplicons. Psyllids reared in healthy *Murraya paniculata* seedling were used as negative control and psyllids previously positive in the qPCR for Las [22] were used as positive control for the presence of Las.

### 2.4. Analysis of the Presence of Candidatus Liberibacter asiaticus in Diaphorina citri

The percentage of psyllids carrying Las was calculated from the number of samples with Las over the total samples analyzed in each fortnight interval.

In addition, the occurrence of psyllids with Las (Las+) among regions and by first (summer/autumn) and second (winter/spring) semesters was transformed using the proportion of Las + psyllids in relation to the total number of analyzed psyllids. The time-period defined was based on: (i) seasonality, summer/autumn and winter/spring; (ii) the period with the presence of shoots V1, V2, and V3 shoots [19]; and (iii) psyllid capture by traps as a result of citrus flushing (Supplementary Figure S1). Thereby, the two compared seasons encompassed the periods of January to June (summer + autumn) and July to December (winter + spring). This analysis was restricted to southwestern (Avaré and Santa Cruz do Rio Pardo), central (Araraquara), and northern (Bebedouro) regions of SP state, due to data availability. The two seasons were compared by a proportion test (*Z* test) at 5% significance [31].

To address the effect of HLB management in the presence of Las in psyllids, psyllids collected between March 2016 and March 2017 were used to calculate the proportion of Las + psyllids, followed by a comparison using the proportion test (Z test) at 5% significance [31]. During this period, two farms with type A management (farms at Iaras and Gavião Peixoto municipalities, SP) were compared with regional data from traps on farm with management types B and C. Farms at Iaras and Gavião Peixoto were located in southwestern (Avaré) and central (Araraquara) regions, respectively.

The same data used in the Z test were used to obtain a temporal series of the proportion of Las + psyllids in relation to the total number of psyllids collected in each period (fortnightly). Spectral analysis identified the frequency of occurrence of principal peaks from the temporal series. It used the sums of trigonometric functions (sine and cosine) to data time series based on the Fourier theorem. This technique identified the most significant cycles [32]. The series were detrended by autocorrelation functions using an Equation (1), where x was the original value in the series, t was the time lag, a was an intercept, and b was the slope. The spectral density estimates were smoothed using a “seven Hamming window” to avoid spurious peaks of temporal patterns. The peaks, originated from the spectral density, were chosen for interpretation; the highest peaks registered in the periodogram were the most important cycles of the studied phenomenon [32]. Data were transformed to reduce noise, tendencies, and non-stationary signals. The main *D. citri* population peak indicated the frequency, in days, that main peaks occurred for Las + psyllids.

xd = x − (a + b × t)(1)

Our analysis comprised psyllids caught in yellow sticky traps in the open environment, from a set of traps covering a range of environments, management strategies, and collection sites. Management type was classified at the beginning of the collection for each year (Supplementary Table S1). There was no audit for the management applied during the whole span of the experiment. Seasonal comparison of both incidences and titer of Las in *D. citri* were not carried out since the traps in each sample point evaluated were not the same during the study.

## 3. Results

### 3.1. Presence of Candidatus Liberibacter asiaticus in Diaphorina citri

Psyllids were sampled from yellow sticky traps at 317 time points. In addition, no psyllids or no data collection were performed at nine time points. The number of psyllids collected varied from 1 to 50, averaging 31 individuals per time point (data not shown). Overall, 10,212 adult psyllids were collected from the traps and 339 samples had Ct values for the *wingless* gene from *D. citri* above the cut-off value and were excluded from analysis. As a result, 9873 samples were employed for analysis, where Las was detected in 6449 psyllids or 65.3% of the individuals (Table 1). Adult individual psyllids analyzed per region ranged from 749 in the northern SP state/southwestern MG state (Frutal) to 3117 in the central region of SP state (Araraquara). There was a gradient in the percentage of psyllids carrying Las; the southwestern region had a mean percentage of 74.5% of psyllids with Las (Avaré and Santa Cruz do Rio Pardo regions); central had 67.1% (Araraquara region) and northern SP had 55.8% (Bebedouro region), while in the northern SP/southwestern MG state had the lowest percentage, with 33.0% (Frutal region). These percentages take into consideration only psyllids caught in traps monitored by Fundecitrus, covering a range of management practices, but mostly on poorly managed areas for HLB, without diseased tree removal and psyllid control or no control at all, management categorized here as B and C. In addition, psyllids caught in yellow sticky traps from two farms with HLB management type A were also evaluated, where percentages of Las + psyllid ranged from 65.7% in southwestern to 58.6% in central regions of SP state.

Our sample data set had Ct values for Las from 13.2 to 35.0, with an average Ct of 27.2. Of the samples, 1.0% had Ct values lower than 15.6, 6.4% had Ct values between 15.7 and 19.1, 12.9% Ct values between 19.2 and 22.6, 19.6% Ct values between 22.7 and 26, 18.7% between 26.1 and 29.4, 23.4% between 29.5 and 32.9 and 19.1% Ct values between 32.9 and 35.0. These values correspond to unit decreases in Log_10_ of 16SrDNA from Las in the psyllid body (titer) [30], starting from values above 6 to less than 1.0 (Table 2). There was a trend to have more samples with titer below 1.0 in the northern regions, while the percentage with the highest titer was higher in the southwestern locations (Avaré and Santa Cruz do Rio Pardo).

Since Ct values from the *wingless* gene from *D. citri* was simultaneously obtained with that of Las, a relative quantification of the abundance of 16SrDNA from the Las copy number in relation to the *wingless* gene *D. citri* copy number would be possible. This would adjust the titer of Las in relation to *D. citri* titer, due to the fact that a wider range of DNA amount is obtained from the sample when the psyllid body is recovered from the trap than when whole and alive bodies are collected (average Ct of 26.2 and range of 16.6 to 36.0 from the traps—the average Ct of 24 for live captured psyllids [30]). Moreover, this analysis was carried out (data not shown). The average distribution of psyllid samples in relation to relative titer was different from the one presented in Table 2, increasing the number of psyllids with higher relative titer of Las (relation closer to 1:1, data not shown).

### 3.2. Seasonality of the Presence of Candidatus Liberibacter asiaticus in Psyllids

**Figure 2:**
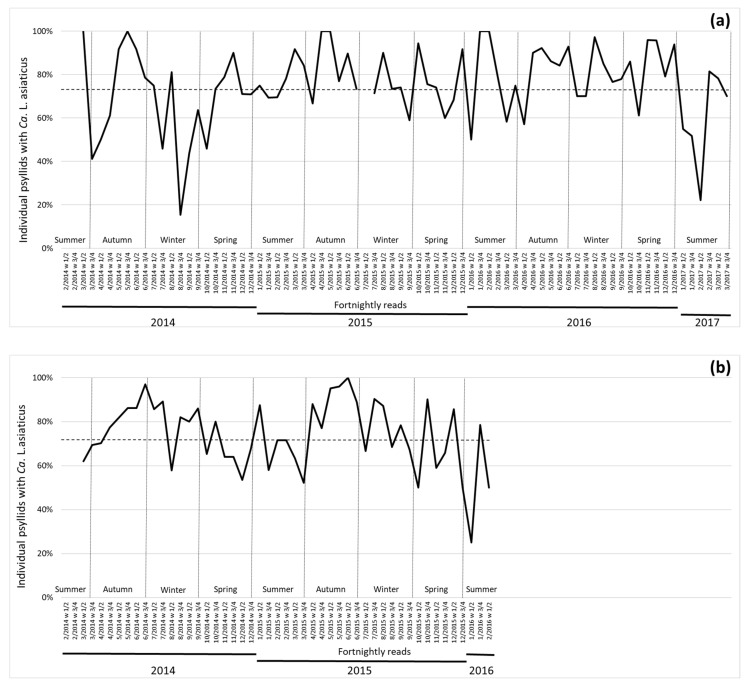
Percentage of psyllids carrying *Candidatus* Liberibacter asiaticus in fortnightly reads between February 2014 to March 2017. Dotted line indicates average Las + psyllid for the region (each time point is indicated with month/year numbering and week interval). Season indication was added to the image according to average dates from the southern hemisphere. (**a**) Avaré region (two time points are missing); (**b**) Santa Cruz do Rio Pardo region (data up to February of 2016), both in southwestern region of São Paulo state.

The two adjacent regions of Avaré and Santa Cruz do Rio Pardo (Figure 1) comprised citrus plantations located in the southwestern of SP state and presented a similar percentage of psyllids with Las, 74.6 and 74.1%, respectively (Figure 2). The lowest percentage of Las found in the Avaré region (15.4%) was in the second half of August 2014 (winter), while in Santa Cruz do Rio Pardo, the lowest value was 25% in January 2016 (summer). Seven time points had 100% psyllids with Las (98 individuals) in the southwestern region, representing the highest values among regions, occurring from January to July (summer/autumn) (Figure 2).

**Figure 3:**
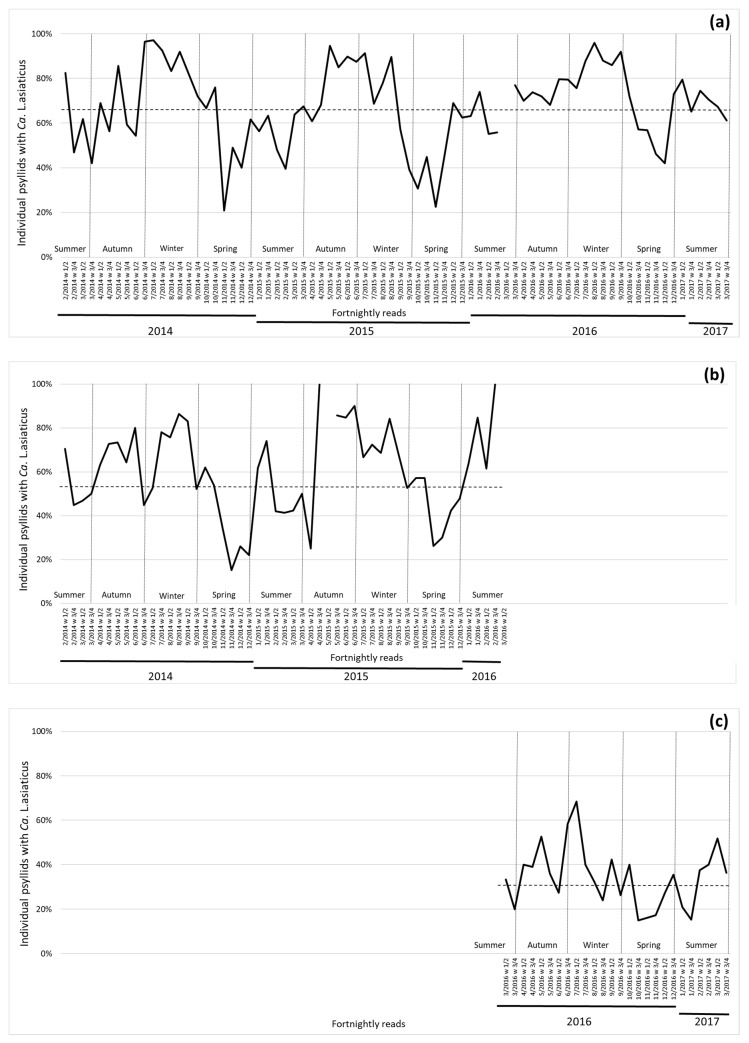
Percentage of psyllids carrying *Candidatus* Liberibacter asiaticus in fortnightly reads between February 2014 to March 2017. Dotted line indicates average for the region (each time point is indicated with month/year numbering and week interval). Season indication was added to the image according to standard occurrence in the southern hemisphere. (**a**) Central region (Araraquara; 1 time point missing); (**b**) northern region (Bebedouro; data up to February of 2016; one time point missing), and (**c**) northern region of São Paulo/southwestern region of Minas Gerais states (Frutal; data starts March of 2016).

The central region (Araraquara) had a slightly lower percentage of psyllids with Las (67.1%), while in northern (Bebedouro), the percentage of psyllids with Las was even lower (55.8%). Both regions presented a typical seasonal pattern, showing a decline in the percentage of Las + psyllids from winter to spring and having a steady increase in the percentage of Las + psyllids from spring to summer/autumn (Figure 3). The Frutal region had the lowest percentage of psyllids with Las (33.0%), following the trend of central and northern regions of SP state in the decrease and increase of the percentage of psyllids carrying Las (Figure 3).

The psyllid population showed an increase in winter and spring seasons, with slight variations among years and regions (Supplementary Figure S1A). This increase followed the flush shoot development period of citrus plants (Supplementary Figure S1B).

### 3.3. Proportion of Candidatus Liberibacter asiaticus in Psyllids

There was a significant difference in southwestern (Santa Cruz do Rio Pardo) and central (Araraquara) regions in 2015 in the seasonal proportion of psyllids with Las, which was higher in summer/autumn (January to June) (Table 3). In the northern region (Bebedouro), no significant seasonal difference was observed. In 2016, in the central (Araraquara) and southwestern (Avaré) regions, no semesterly differences were observed but both had an increase in the proportion of Las + psyllids (Table 3). The higher proportion of psyllids carrying Las occurred in the southwestern region, irrespective of the locations and with punctual impact of seasonality.

The occurrence of psyllids with Las among regions in the same time period (January to June and July to December; 2014 to 2016) showed that northern SP (Bebedouro region) was consistently lower than southwestern (Avaré and Santa Cruz do Rio Pardo regions) (Table 4). The northern region also had a lower proportion in comparison with central SP (Araraquara region) from July 2014 to June 2015. In the semester of July to December 2015, no difference was found, mainly because the proportion of Las + psyllids was lower in the central region. With the exception of the semester from July to December 2014, central and southwestern regions differed in the proportion of Las + psyllids, with lower values in the central region. A higher proportion of Las + psyllids was found in the southwestern region, reaching the highest value of 0.83 in July to December 2016 (Table 4).

### 3.4. HLB Management Comparison Study

**Figure 4:**
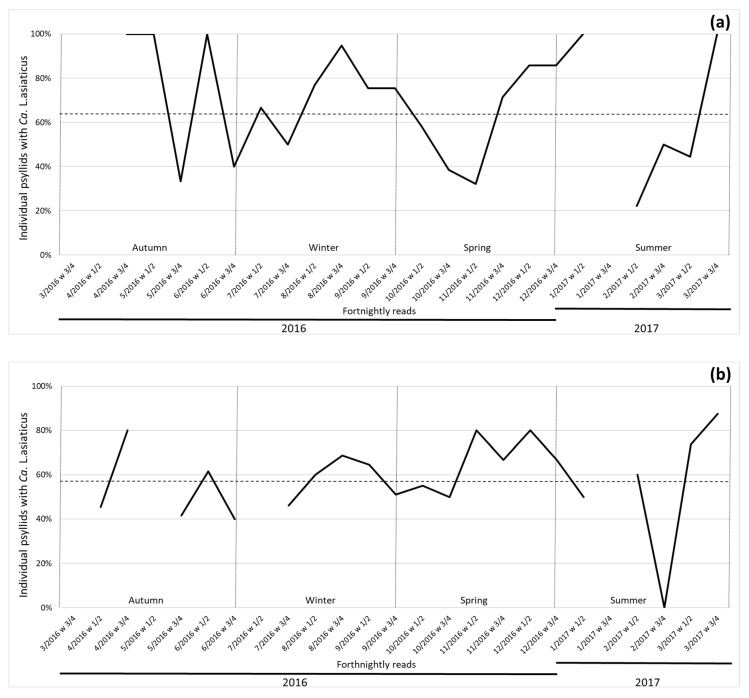
Percentage of psyllids carrying *Candidatus* Liberibacter asiaticus in fortnightly reads between March 2016 to March 2017. Dotted line indicates average for the farm (each time point is indicated with month/year numbering and week interval). Season indication was added to the image according to standard occurrence in southern hemisphere. (**a**) Iaras from the southwestern region of Avaré (two time points missing); (**b**) Gavião Peixoto from the central region in Araraquara (data up to February of 2016; three time points missing).

The trend of psyllids carrying Las was similar when data from good management farms were compared with the region they belong to (Figure 4). However, psyllids collected from two farms with good management practices had a lower percentage of Las when compared with the regions they belong to; one farm from the municipality of Iaras had 65.7% of the psyllids with Las, while in Avaré, the region where this municipality is located, the average percentage was 74.6% (*p* = 0.0006). The farm from Gavião Peixoto had 58.6% of psyllids with Las and the Araraquara region had an average of 67.1% (*p* = 0.0001). When comparing the number of Las + psyllids between citrus farms with good HLB management practices (Iaras and Gavião Peixoto) and poor management practices (Avaré and Araraquara regions, respectively), Iaras had an average of 15% fewer psyllids with Las than the Avaré region. In the case of Gavião Peixoto, the average was 11.3% lower than the Araraquara region.

Additionally, the number of Las + psyllid main peaks indicated by spectral densities showed differences in the central region; orchards with bad management practices had twice the number of Las + psyllid occurrence main peaks than ones with good management practices (eight versus four occurrences of main peaks, respectively). The same was observed in the southwestern region with three versus seven occurrences of main peaks for good versus bad management, respectively.

## 4. Discussion

We conducted an extensive investigation of the presence of Las in *D. citri* adults caught on yellow sticky traps, assessing the percentage of individuals carrying Las in relation to regions of the Brazilian citrus belt (SP and MG states), season of the year, and to rigorous or weak HLB management. In this study, the overall infection frequency of Las in psyllids caught in yellow sticky traps was 65.3% (6449/9873). DNA quality and amount would be a concern for detection and quantification of *Ca.* Liberibacter spp. in the psyllid body when caught in the traps in the open environment, especially in hot and humid conditions. However, in only 3.3% of the sampled psyllids, the *wingless* gene from *D. citri* was not detected with qPCR or with Ct values above the cut-off. We used *wingless* detection as a parameter to evaluate the quality and quantity of the template DNA [23] and the samples lacking the quality necessary for analysis were discarded. Detection of *Ca*. Liberibacter spp. in psyllids caught on yellow sticky traps in similar situations was done before [13]. No negative effect on Las detection was observed in psyllids exposed to the environment for up to 15 days in such traps [33]. The observed loss rate might be related to sample degradation in the environment due to various reasons, loss of DNA during extraction, or another insect caught instead of the psyllid *D. citri*. This might be a disadvantage to the use of psyllids caught in yellow sticky traps when compared with live capture using suction devices [26,27]. However, with the averages of psyllids found in citrus orchards in Brazil, particularly in orchards under psyllid management, yellow sticky traps represent a valuable source to monitor not only psyllid presence [14] but also Las occurrence [13].

While qPCR detects the presence of Las in psyllids, that does not necessarily mean all psyllids are able to transmit Las, since the bacterium may have been recently acquired and is still in its latency period [34] or has not achieved the circulative stage or enough titer to be inoculated [35]. The threshold infection density of *circa* 10^6^ Las gene copies per insect represented 8.5% of psyllids able to inoculate Las in citrus trees, even though Las was detected in 99.4% of the psyllids [35]. Live adult psyllids were collected by suction, raised from nymphs in citrus with HLB symptoms [35]. By measuring Las titer in adults [30] and taking a similar titer threshold into consideration for our population of 6449 Las + psyllids, would result in 1.0% of samples having this competency. Adding 6.4% of psyllids that have between 10^5^ to 10^6^ Las gene copies, to account for less DNA obtained from samples collected from traps than when using live individuals, a more predictive value of 7.4% infective adults would be achieved, similar to that observed in Japan [35]. If this is a true threshold in tropical and subtropical conditions, like the ones evaluated in this work, it still merits validation. A relation of 1:1 between Las and *D. citri* gene copy number was obtained for psyllids heavily infected with Las [36]. The relative quantification of Las 16SrDNA copy number over the *D. citri wingless* copy number would provide an accurate quantification of Las titer [20]. The utilization of absolute quantification for both Las [37] and psyllid copy number provided by droplet digital PCR may allow more accurate quantification of Las titer. The relationship with transmission assays would be a step forward to understand HLB epidemiology.

Averages of Las + psyllids per region were higher in the southwestern region of SP (74.5%) decreasing continuously from central to northern SP (67.1% and 55.8%, respectively) and even decreasing more at the SP/MG state border (33%). Furthermore, not only the increment in psyllids caught but also the percentage of Las + psyllid followed the sprouting period, starting at spring and increasing further in summer and autumn, particularly in the central and northern regions of the Brazilian citrus belt. Winter is the season of highest Las incidence in Colima, Mexico [26] and Florida, USA [27], being the season of high prevalence of Las + psyllids in central (SP) and northern regions (SP/MG). In the southwestern region, the steady and high prevalence of Las + psyllids had no seasonality, although autumn had the higher percentages. There is a temperature gradient, higher for MG/northern SP, intermediate in the center of SP, and lower in southwestern region of SP state, with temperature and rainfall levels being phenological determinants [30,38]. There was an increase in the percentage of Las + psyllids starting at mid-spring in the central and northern regions, following the spring flush. Spring to summer are the seasons of higher occurrence of *D. citri* in central and northern São Paulo state [39] and an increase in the population of this insect vector occurs after the flush stage and with an increase in the temperature. Thus, high psyllid populations with an increase in the incidence of Las render a higher risk of primary infections in citrus orchards.

In Colima, Mexico, the proportion of positive psyllids (Las + psyllids) tend to be higher in lower temperatures (winter/spring), while the opposite was observed with the increase of temperature of summer [26]. However, the proportion of positive psyllids was not statistically different among seasons [26]. In Florida, USA, the incidence of Las in *D. citri* was more pronounced during late autumn and early winter than in mid- to late summer, with an effect of air temperature on this incidence [27]. Temperature had an influence on titer of *Ca.* Liberibacter spp. [40] and this reflects in Las acquisition rates by *D. citri* [30,41] and in the incidence of Las in psyllids [42]. The higher the temperature, the lower the acquisition and incidence of Las in *D. citri*.

Our results allowed us to connect the Las + psyllid data to different citrus regions, its HLB incidence, and management practices. For instance, Las acquisition by adult psyllids and the titer of Las in both psyllid and citrus were similar between farms located in the central region of SP and southwestern MG [41]. However, a comparison between two farms in the southwestern region of MG, differed in the same parameters [41]. These locations were included in our study, reflecting microclimates occurring in large, regional areas evaluated here. Additionally, a large sampling assessment revealed similarities between southwestern regions of Santa Cruz do Rio Pardo and Avaré, where trends and percentage of Las + psyllids were similar. Such a high incidence of psyllids with Las in the southwestern region of SP state since 2014, preceded the increase in HLB in that region, with the following HLB incidences from 2015 to 2020: 4.4, 8.0, 9.8, 10.8, and 16.8%, respectively [15]. From the other side, for the same years, in the central region of SP, HLB incidences were high with the tendency to drop in the last three years: 19.9, 17.6, 21.6, 18.3, 17.3, and 14.5%, respectively, while in the southwestern MG/northern SP, the incidence was consistently low: 0.08, 1.07, 0.58, 0.0, 0.31, and 0.08%, from 2015 to 2020, respectively. There is evidence that warmer temperatures affect the titer of Las in plants, having an influence on Las acquisition by psyllids and even in the spread of the disease [30,41,42]. In central and northern regions, the trend of Las + psyllid was similar, with an increase starting from the spring to autumn, but with differences that reflect the HLB incidence in those regions. Although our data preclude statistical analysis of this relationship, it is a similar situation where growers look at where “hot” psyllids, i.e., Las + psyllids, are spreading and entering the farm to search for reservoirs of HLB-affected trees or sites for Las acquisition by psyllids [43]. In the case of citrus blocks or citrus farms, the edge effect is a well know phenomenon [2,12,44] and it seems to be the case with psyllids in large, regional assessments as well. The central region of SP is where HLB was first found in Brazil in 2004 [5,6] in a very conductive region for HLB [41] but where growers are continuously using better HLB management practices [2,11,12,13]. Current HLB incidence levels in SP and MG are 20.87% of the ~200 million trees [15].

The comparison of poor management versus good management areas, with lower percentage of psyllids with Las when doing symptomatic tree eradication and psyllid control, reflect the strategy of wide area management, that found 10 times less Las + psyllids in the citrus block with good management practices nearby, in comparison with blocks with poorly managed groves in the vicinity of the experimental area [13]. While the difference in the current study was about 10% less psyllids with Las in well managed farms compared to poorly managed areas, actions to reduce HLB presence outside the farm is reflected in the primary spread at the edges of well managed areas. This is the so-called external HLB management, where growers seek symptomatic trees outside orchards in a radius of 3, 5, or 7 km of the citrus commercial property [28]. When symptomatic trees are removed, the source of inoculum for psyllids to acquire the bacterium when feeding outside the managed orchard is reduced. Alternatively, in the case that they feed inside the orchard, a higher proportion of psyllids are free of Las and even if they lay eggs, their offspring are grown on a non-inoculated plant, emerging as healthy adult psyllids [21]. While a number of factors might affect the incidence of Las in *D. citri* [27;35], HLB management also may have an influence as well [13].

## 5. Conclusions

*Ca*. L. asiaticus incidence in *D. citri* in Brazil were on average 65.3% of the individuals sampled in the interval between 2014 and 2017. There was a higher proportion of Las + psyllids from January to June (summer and autumn) than from July to December (winter and spring) in the southwestern and central SP regions in 2015. Shoot sprout is followed by an increase in the psyllid population measured as captures in yellow sticky traps, with an increase in Las + psyllids starting in the spring in central and northern regions. Good local and area-wide HLB management practices reduce the percentage of psyllids with Las.

## Figures and Tables

**Table 1:** Locations and period where yellow sticky traps were monitored for the presence of psyllids and sampled for *Candidatus* Liberibacter asiaticus detection.

Region (Name) ^a^	Sampling Period	Traps as Source of Psyllids ^b^	Psyllids Analyzed/Sampled ^c^	Psyllids with Las (%) ^d^
Southwestern (Avaré)	February 2014 to March 2017	Fundecitrus	1910/2025	1425 (74.6)
Southwestern (Santa Cruz do Rio Pardo)	February 2014 to February 2016	Fundecitrus	1940/2000	1438 (74.1)
Central (Araraquara)	February 2014 to March 2017	Fundecitrus	3117/3210	2092 (67.1)
Northern (Bebedouro)	February 2014 to February 2016	Fundecitrus	1427/1459	796 (55.8)
Northern (Frutal)	March 2016 to March 2017	Fundecitrus	749/766	247 (33.0)
Southwestern (Iaras)	March 2016 to March 2017	Grower	324/338	213 (65.7)
Central (Gavião Peixoto)	April 2016 to March 2017	Grower	406/414	238 (58.6)
Total (average %)			9873/10,212	6449 (65.3)

**^a^** Regions are classified in relation to the overall location of the citrus belt in São Paulo (SP) and Minas Gerais (MG) states, with the name of the region stated in parenthesis. ^b^ Yellow sticky traps from Fundecitrus were located in citrus orchards or backyards with no removal of diseased trees and with or without psyllid control (management B or C). Yellow sticky traps from growers were in citrus orchards with removal of diseased trees and psyllid control (management A). **^c^** All psyllids samples had DNA extracted individually (sampled), while only samples with Ct values below or equal to 36.0 (DCp) were considered for analysis (analyzed). **^d^** Samples whose Ct values were below or equal to 35.0 (HLBaspr) were considered positive for the presence of *Ca*. L. asiaticus (Las) and the percentage was calculated in relation to samples analyzed.

**Table 2:** Titer of *Candidatus* Liberibacter asiaticus in individual adult psyllids (*Diaphorina citri*) sampled in yellow sticky traps as determined by qPCR in relation to sampled regions.

Ct Range (HLBaspr) Range (Titer) ^a^	Southwestern (Avaré) ^b^	Southwestern (S. C. R Pardo)	Central (Araraquara)	Northern (Bebedouro)	Northern (Frutal)	Southwestern (Iaras)	Central (Gavião Peixoto)	Overall
<15.6 (>6.1)	22 (1.5%)	20 (1.4%)	14 (0.7%)	5 (0.6%)	2 (0.8%)	-	-	63 (1.0%)
15.7 to 19.1 (5.1 to 6.0)	109 (7.6%)	157 (10.9%)	148 (7.1%)	64 (8.0%)	10 (4.0%)	2 (0.9%)	-	490 (6.4%)
19.2 to 22.6 (4.1 to 5.0)	202 (14.2%)	207 (14.4%)	307 (14.7%)	103 (12.9%)	33 (13.4%)	17 (8.0%)	31 (13.0%)	900 (12.9%)
22.7 to 26.0 (3.1 to 4.0)	227 (15.9%)	261 (18.2%)	384 (18.4%)	116 (14.6%)	54 (21.9%)	50 (23.5%)	59 (24.8%)	1151 (19.6%)
26.1 to 29.4 (2.1 to 3.0)	286 (20.1%)	301 (20.9%)	405 (19.4%)	132 (16.6%)	34 (13.8%)	42 (19.7%)	49 (20.6%)	1249 (18.7%)
29.5 to 32.9 (1.1 to 2.0)	341 (23.9%)	303 (21.1%)	469 (22.4%)	192 (24.1%)	53 (21.5%)	62 (29.1%)	52 (21.8%)	1472 (23.4%)
33.0 to 35.0 (0.4 to 1.0)	238 (16.7%)	189 (13.1%)	365 (17.4%)	184 (23.1%)	61 (24.7%)	40 (18.8%)	47 (19.7%)	1124 (19.1%)
Sum	1425	1438	2092	796	247	213	238	6449

**^a^** Ct values range were based on titer quantification using equation for the copy number of 16SrDNA from Las [30]. ^b^ In each region, the evaluated number of samples with Ct values in the range is presented, with the percentage in relation to the region in parentheses.

**Table 3:** Proportion of psyllids carrying *Candidatus* Liberibacter asiaticus (Prop. Las +) in comparison between semesterly periods inside regions.

Period/Region	Southwestern (Avaré) ^a^		Southwestern (Santa Cruz do Rio Pardo)		Central (Araraquara)		Northern (Bebedouro)	
	Prop. Las+	*p*-Value	Prop. Las+	*p*-Value	Prop. Las+	*p*-Value	Prop. Las+	*p*-Value
15 January to 15 June	0.79a	0.2858	0.79a	0.0041	0.67a	0.0003	0.56a	0.6192
15 July to 15 December	0.75a		0.71b		0.56b		0.54a	
16 January to 16 June	0.82a	0.7674	-	-	0.70a	0.3169	-	-
16 July to 16 December	0.83a			-	0.73a		-	

^a^ In each region, *p*-values lower than 0.05 and prop. Las + followed by different letter between two consecutive semesters indicates significant differences between psyllids carrying Las (January to June and July to December). - Indicates no data available.

**Table 4:** Comparison of the proportion of psyllids carrying *Candidatus* Liberibacter asiaticus among southwestern, central, and northern regions of São Paulo state, Brazil and the observed *p* value for each combination. ^a^

**July to December/2014 ^b^**				
	Southwestern-Avaré (0.66)	Southwestern-Santa Cruz (0.74)	Central-Araraquara (0.68)	Northern-Bebedouro (0.48)
Southwestern-Avaré (0.66)	-			
Southwestern-Santa Cruz (0.74)	0.0066	-		
Central-Araraquara (0.68)	0.5056	0.0294	-	
Northern-Bebedouro (0.48)	0.0001	0.0001	0.0001	-
**January to June/2015**				
	Southwestern-Avaré (0.79)	Southwestern-Santa Cruz (0.79)	Central-Araraquara (0.67)	Northern-Bebedouro (0.56)
Southwestern-Avaré (0.79)	-			
Southwestern-Santa Cruz (0.79)	1	-		
Central-Araraquara (0.67)	0.0007	0.0001	-	
Northern-Bebedouro (0.56)	0.0001	0.0001	0.0020	-
**July to December/2015**				
	Southwestern-Avaré (0.75)	Southwestern-Santa Cruz (0.71)	Central-Araraquara (0.56)	Northern-Bebedouro (0.54)
Southwestern-Avaré (0.75)	-			
Southwestern-Santa Cruz (0.71)	0.2535	-		
Central-Araraquara (0.56)	0.0001	0.0001	-	
Northern-Bebedouro (0.54)	0.0001	0.0001	0.5715	-
**January to June/2016 ^c^**				
	Southwestern-Avaré (0.82)	Central-Araraquara (0.70)		
Southwestern-Avaré (0.82)	-			
Central-Araraquara (0.70)	0.0034	-		
**July to December/2016 ^c^**				
	Southwestern-Avaré (0.83)	Central-Araraquara (0.73)		
Southwestern-Avaré (0.83)	-			
Central-Araraquara (0.73)	0.0001	-		

^a^ In each period, *p* values lower than 0.05 between the regions indicate significant differences between the proportion of psyllids carrying Las (July/December and January/June). ^b^ Values inside parenthesis indicate the proportion of psyllids carrying Las for each region. ^c^ Periods without data for southwestern (Santa Cruz = Santa Cruz do Rio Pardo) and northern (Bebedouro).

## Supplementary Materials

The following are available online at https://www.mdpi.com/2075-4450/11/10/672/s1, Table S1. Locations and number of traps belonging to Fundecitrus Psyllid Alert System, monitored by Fundecitrus or by the growers fortnightly in the sampled period; Table S2. Information of GPS points from traps used in this study; Figure S1: A. Percentage of citrus plants with flush shoots at stages V1+V2+V3 evaluated simultaneously with psyllid capture; B. Average psyllids caught on yellow sticky traps. Both comprised the whole data set from the Fundecitrus Alert System at the indicated fortnightly time periods for each year.
