# Supplementary material for: Incidence of Diaphorina citri Carrying Candidatus Liberibacter asiaticus in Brazil’s Citrus Belt
Source: Insects. 2020 Oct 3;11(10):672. doi: 10.3390/insects11100672 (PMC7650542; doi:10.3390/insects11100672)
Supplement: Supplementary file 1 [file insects-11-00672-s001.zip › Figure S1.docx]

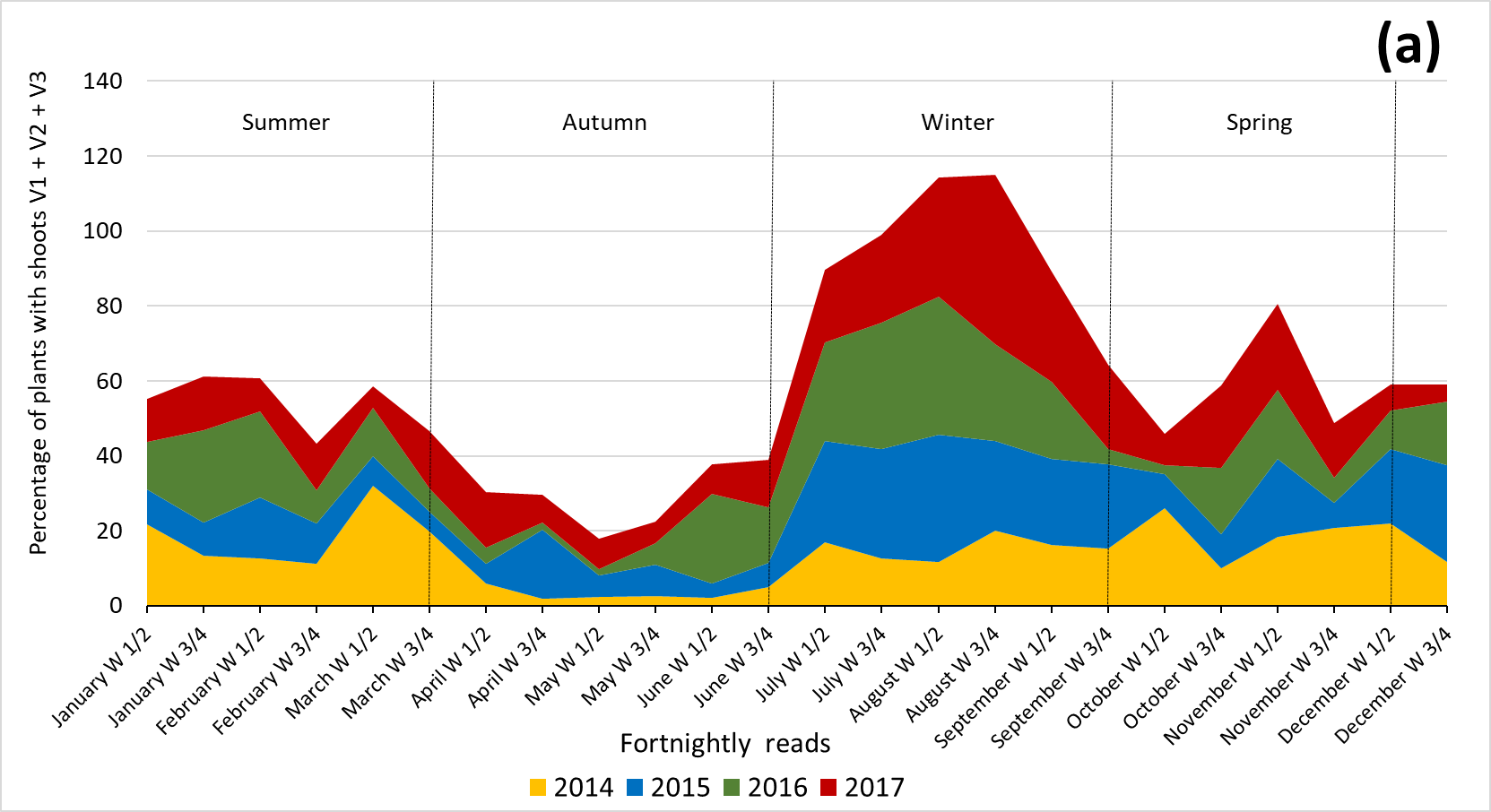


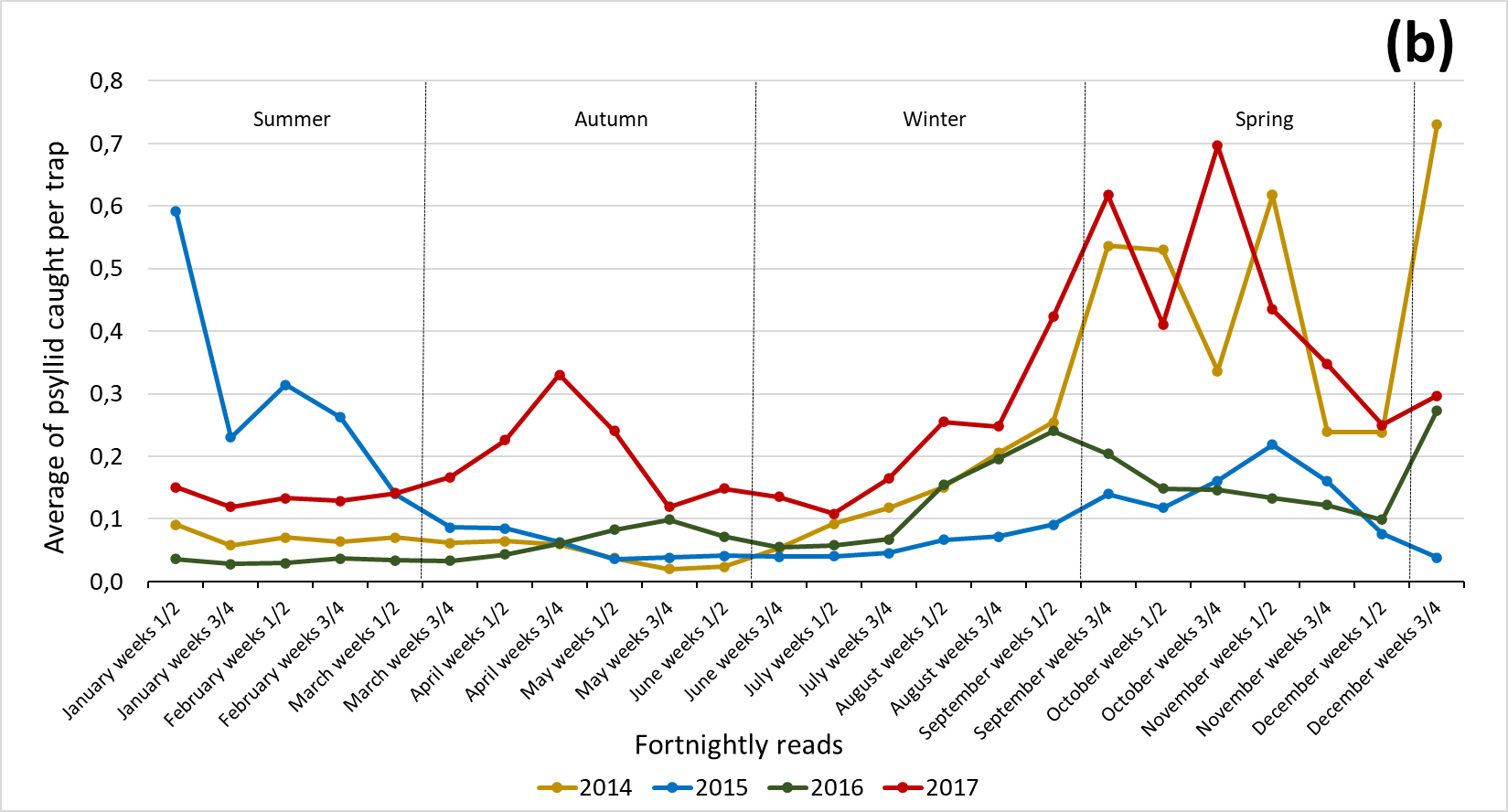


**Figure S1**. **A.** Percentage of citrus plants with flush shoots at stages V1+V2+V3 evaluated simultaneously with psyllid capture; **B.** Average of psyllids caught on yellow sticky traps. Both comprised the whole data set from the Fundecitrus Alert System at the indicated fortnightly time periods for each year.
