# Supplementary material for: Incidence of Diaphorina citri Carrying Candidatus Liberibacter asiaticus in Brazil’s Citrus Belt
Source: Insects. 2020 Oct 3;11(10):672. doi: 10.3390/insects11100672 (PMC7650542; doi:10.3390/insects11100672)
Supplement: Supplementary file 1 [file insects-11-00672-s001.zip › Table S1.docx]

**Table S1**. Locations and average number of yellow sticky traps belonging to Fundecitrus Psyllid Alert System, fortnightly monitored by Fundecitrus or by citrus growers in the sampled period.

| **Region name** | **Municipality ^a^** | **Yellow sticky traps ^b^** | |
| --- | --- | --- | --- |
|  |  | **Fundecitrus** | **Growers** |
| February 2014 to February 2015. | | | |
| Southwestern - Avaré | Angatuba, Anhembi, Arandú, Avaré, Bofete, Borebi, Botucatu, Buri, Cerqueira César, Coronel Macedo, Iaras, Itaberá, Itaí, Itapeva, Itapetininga, Itatinga, Lençóis Paulista, Manduri, Paranapanema, Pardinho, Piracicaba, Pratânia e Taquarituba (São Paulo State - SPS) | 298 | 1,377 |
| Southwestern - Santa Cruz do Rio Pardo | Águas de Santa Bárbara, Agudos, Avaí, Cabrália Paulista, Duartina, Espírito Santo do Turvo, Fernão, Gália, Lucianópolis, Óleo, Paulistânia, Santa Cruz do Rio Pardo, São Pedro do Turvo e Ubirajara (SPS) | 232 | 1,951 |
| Central - Araraquara | Américo Brasiliense, Analândia, Araraquara, Boa esperança do Sul, Borborema, Brotas, Gavião Peixoto, Ibaté, Ibitinga, Itaju, Itápolis, Matão, Nova Europa, Ribeirão Bonito, Reginópolis, Rincão, São Carlos, Tabatinga e Taquaritinga (SPS) | 377 | 1,667 |
| Northern - Bebedouro | Altair, Barretos, Bebedouro, Cajobi, Colina, Colômbia, Guaraci, Icem, Monte Azul Paulista, Nova Granada, Olímpia, Onda Verde, Palestina, Severínia, Taquaral e Viradouro (SPS) | 288 | 4,675 |
| March 2015 to February 2016. | | | |
| Southwestern - Avaré | Angatuba, Anhembi, Arandú, Avaré, Borebi, Botucatu, Buri, Capão Bonito, Cerqueira Cesar, Cesário Lange, Coronel Macedo, Iaras, Itaberá, Itaí, Itapetininga, Itapeva, Itatinga, Lençóis Paulista, Manduri, Pardinho, Piracicaba, Pratânia, Santa Maria da Serra, São Manuel, São Miguel Arcanjo, Taquarituba, Taquarivaí e Tatuí (SPS) | 294 | 2,840 |
| Southwestern - Santa Cruz do Rio Pardo | Águas de Santa Bárbara, Agudos, Alvinlândia, Cabrália Paulista, Duartina, Espirito Santo do Turvo, Fernão, Gália, Lucianópolis, Lupércio, Ocauçu, Óleo, Paulistânia, Piratininga, Santa Cruz do Rio Pardo, São Pedro do Turvo e Ubirajara (SPS) | 186 | 1,792 |
| Central - Araraquara | Américo Brasiliense, Analândia, Araraquara, Bariri, Boa Esperança do Sul, Brotas, Gavião Peixoto, Ibaté, Ibitinga, Itaju, Itirapina, Luís Antonio, Matão, Motuca, Nova Europa, Ribeirão Bonito, Rincão, São Carlos, São Simão, Tabatinga e Trabiju (SPS) | 343 | 1,860 |
| Northern - Bebedouro | Barretos, Bebedouro, Cajobi, Colina, Embaúba, Guapiaçu, Monte Azul Paulista, Novais, Olímpia, Paraíso, Pirangí, Severínia, Tabapuã, Taiuva, Taquaral, Viradouro e Vista Alegre do Alto (SPS) | 260 | 4,498 |
| March 2016 to March 2017. | | | |
| Southwestern - Avaré | Angatuba, Anhembi, Arandú, Avaré, Borebi, Botucatu, Buri, Capão Bonito, Cerqueira Cesar, Cesário Lange, Coronel Macedo, **Iaras**, Itaberá, Itaí, Itapetininga, Itapeva, Itatinga, Lençóis Paulista, Manduri, Pardinho, Piracicaba, Pratânia, Santa Maria da Serra, São Manuel, São Miguel Arcanjo, Taquarituba, Taquarivaí e Tatuí (SPS) | 213 | 3,148 |
| Central - Araraquara | Américo Brasiliense, Analândia, Araraquara, Bariri, Boa Esperança do Sul, Brotas, **Gavião Peixoto**, Ibaté, Ibitinga, Itápolis, Itaju, Itirapina, Luís Antonio, Matão, Motuca, Nova Europa, Ribeirão Bonito, Rincão, São Carlos, São Simão, Tabatinga e Trabiju (SPS) | 221 | 1,678 |
| Northern - Frutal | Altair, Colômbia, Guaraci, Icém, Nova Granada, Onda Verde, Palestina (SPS),  Água Comprida, Campina Verde, Campo Florido, Comendador Gomes, Conceição das Alagoas, Fronteira, Frutal, Itapagipe, Ituiutaba, Monte Alegre de Minas, Pirajuba, Planura, Prata, São Francisco de Sales, Uberaba, Uberlândia e Veríssimo (Minas Gerais State) | 188 | 3,284 |

**^a^** Municipalities in bold where farm with HLB management type A had psyllids sampled (2016/2017).

**^b^** Psyllids sampled from Fundecitrus’ yellow sticky traps were used to assess *Ca.* L. asiaticus presence by qPCR.
